# Linear-time protein 3-D structure searching with insertions and deletions

**DOI:** 10.1186/1748-7188-5-7

**Published:** 2010-01-04

**Authors:** Tetsuo Shibuya, Jesper Jansson, Kunihiko Sadakane

**Affiliations:** 1 Human Genome Center, Institute of Medical Science, University of Tokyo 4-6-1 Shirokanedai, Minato-ku, Tokyo 108-8639, Japan; 2 Ochanomizu University, 2-1-1 Ohtsuka, Bunkyo-ku, Tokyo 112-8610, Japan; 3 National Institute of Informatics, 2-1-2 Hitotsubashi, Chiyoda-ku, Tokyo 101-8430, Japan

## Abstract

**Background:**

Two biomolecular 3-D structures are said to be similar if the RMSD (root mean square deviation) between the two molecules' sequences of 3-D coordinates is less than or equal to some given constant bound. Tools for searching for similar structures in biomolecular 3-D structure databases are becoming increasingly important in the structural biology of the post-genomic era.

**Results:**

We consider an important, fundamental problem of reporting all substructures in a 3-D structure database of chain molecules (such as proteins) which are similar to a given query 3-D structure, with consideration of indels (*i.e.*, insertions and deletions). This problem has been believed to be very difficult but its exact computational complexity has not been known. In this paper, we first prove that the problem in unbounded dimensions is NP-hard. We then propose a new algorithm that dramatically improves the average-case time complexity of the problem in 3-D in case the number of indels *k *is bounded by a constant. Our algorithm solves the above problem for a query of size *m *and a database of size *N *in average-case *O*(*N*) time, whereas the time complexity of the previously best algorithm was *O*(*Nm*^*k*+1^).

**Conclusions:**

Our results show that although the problem of searching for similar structures in a database based on the RMSD measure with indels is NP-hard in the case of unbounded dimensions, it can be solved in 3-D by a simple average-case linear time algorithm when the number of indels is bounded by a constant.

## Background

It is widely known that biomolecules with similar 3-D structures tend to have similar functions, and we can estimate molecular functions by searching for structurally similar molecules from 3-D structure databases of biomolecules. Thus, to identify similar structures in a biomolecular database is a fundamental task in structural biology [1-5]. Due to recent technological evolution of molecular structure determination methods such as NMR (Nuclear Magnetic Resonance) and X-ray crystallography, more and more structures of biomolecules, especially proteins, are solved, as shown in the increase of the size of the PDB (Protein Structure Data Bank) database [6]. For example, the number of entries in PDB was only around 1000 in 1993 but over 60, 000 in October 2009, and currently grows by about 20% per year. Moreover, a huge number of molecular structures have recently been predicted by various computational techniques. Hence, faster searching techniques against these molecular structure databases are seriously needed. A protein structure is often represented by a sequence of 3-D coordinates that represents the positions of amino acids. Usually, the 3-D coordinates of the *C*_*α *_atom in each amino acid is used as the representative position of that amino acid. Note that there are also other important chain molecules in living cells, such as DNAs, RNAs, and glycans. In this paper, we consider a problem of searching for similar structures from a structure database of chain molecules, which consists of sequences of 3-D coordinates that represent molecular structures.

A tremendous number of algorithms for comparing/searching protein structures have been developed [1-5], which can be categorized roughly into two types. One is a group of algorithms that compare two structures geometrically in the 3-D space, considering the coordinates of structures [7-13] as their inputs. They assume that the structures are rigid or near-rigid, and superimpose (substructures of) the two structures by rotating and translating one of them. The other is a group of algorithms that use more abstract information of the structures, such as the secondary structure elements (SSEs) [14-17]. In this paper, we focus on the first type of algorithms, *i.e.*, we compare the sequences of coordinates without any abstraction. To compare two structures, we need a way to measure their similarities. The most widely-used geometrical similarity measure between two molecular structures is the RMSD (Root Mean Square Deviation) [5,18-23]. There are also many other measures, but many of them are just variants of the RMSD [4]. The RMSD is also used in various other fields, such as robotics and computer vision. It is defined as the square root of the minimum value of the average squared distance between each pair of corresponding atoms, over all the possible rotations and translations. (See the preliminaries section for more details.) The RMSD measure corresponds to the Hamming distance in the textual pattern matching, from the viewpoint that it does not consider any indels (*i.e.*, insertions and deletions) between them. The RMSD can be computed very easily if we are given the correspondence of the atoms (see the preliminaries section), like in the case of computing the Hamming distance.

In the case of textual bio-sequence comparison (such as comparison of 1-D protein sequences), we often prefer to use the string alignment score that takes indels into account in comparing two bio-sequences, rather than to use the Hamming distance. Likewise, it is also important to consider indels when we compare two molecular 3-D structures. In fact, most structural alignment algorithms consider indels. (Note that some of the structural alignment algorithms ignore the order of the atoms on the backbone, but we do not change the order of the atoms in this paper.) But it is much harder than the textual string cases to compare two 3-D structures with consideration of indels, though an ordinary pairwise alignment algorithm for textual strings requires only quadratic time. It has been believed to be almost impossible to compute the alignment that optimizes the RMSD measure. In fact, almost all the previous structural alignment/comparison/searching algorithms that take indels into account are heuristic.

But there have been only a few theoretical results on the difficulty of the structural alignment/comparison/searching problems. Goldman et al. [24] showed that the contact map problem is NP-hard. They formulate the structural alignment problem as a maximization problem on a graph, without considering the structural similarity measures like the RMSD. Zhu [25] showed that the structure alignment problem under a measure called 'discrete Fréchet distance' is also NP-hard. Lathrop [26] showed that the protein threading problem is also NP-hard, but it is not a problem of comparing two molecular structures, but a problem of comparing a molecular structure with a textual sequence of residues. Bu et al. [27] and Shatsky et al. et al [28] showed that several problem formulations of structural motif detection are NP-hard. But none of the above proofs show the NP-hardness of any formulation of structural alignment/comparison problems based on the RMSD measure. It has been a long open problem.

In this paper, we consider a problem of searching for *all *the substructures of database structures whose RMSDs to a given query is within some constant, permitting indels. Though our problem is one of the most straightforward problem formulation for protein structure comparison/alignment/searching, its difficulty is not known. In this paper, we show that our problem is NP-hard if the dimension of the problem is arbitrary.

But it does not mean that our problem is always difficult. If the number of indels is at most some constant, the problem can be solved in polynomial time, though the time complexity of known algorithms is still very large. The best-known algorithm for the problem is a straightforward algorithm that requires *O*(*Nm*^*k*+1^) time for a database of size *N *and a query of size *m*, where *k *is the maximum number of indels. It is the worst-case time complexity, but the average-case (expected) time complexity of the algorithm is still all the same *O*(*Nm*^*k*+1^). We propose in this paper a much faster algorithm that runs in average-case *O*(*N*) time, assuming that the database structures follow some model of molecular physics. We do not mean that the time complexity is against some 'average' structure, but it is the average-case (or expected) time complexity against *all *the possible structures whose distribution follows the model. Unlike most other structural alignment algorithms, our algorithm is not a heuristic algorithm, *i.e.*, our algorithm enumerates *all *the substructures in the database whose RMSD is less than some given bound, permitting a constant number of indels. It means that we cannot achieve better accuracy as long as we use the RMSD as a measure of the accuracy. The worst case time complexity of our algorithm is the same as previous best-known algorithm, *i.e.*, *O*(*Nm*^*k*+1^), whether or not the structures follow any model. Even if the structures do not follow any statistical model, our algorithm outputs accurate results.

The model that we assume against the database structures is a model called the 'random-walk model' (also called the 'freely-jointed chain model' or just the 'ideal chain model'). In the model, the structures are assumed to be generated by random walks. The model is very often used in molecular physics [29-32]. It is also used in the analysis of algorithms for protein structure comparison [10]. As demonstrated in [10], theoretical analyses based on the random-walk model have high consistency with the actual experimental results on the PDB database. Note that our algorithm also runs in linear time if the query structure follows the random-walk model, instead of the database structures.

The organization of this paper is as follows. 'Preliminaries' section describes the notations used in this paper and previous related work as preliminaries. 'The *k*-Indel 3-D Substructure Search Problem' section describes the problem that we solve. 'An NP-Hardness Result' section describes the NP-hardness of our problem. 'The New Average-Case Linear Time Algorithm' section describes our new algorithm and the computational time analysis of the algorithm. 'Conclusions' section concludes our results and discusses the future work.

## Preliminaries

### Notations and Definitions

A chain molecule **S **whose *i*-th 3-D coordinates (vector) is  is noted as **S **= (). The length *n *of **S **is denoted by |**S**|. A structure **S**[*i*..*j*] = () (1 ≤ *i *≤ *j *≤ *n*) is called a *substructure *of **S**. A structure **S***' *= () (1 ≤ *a*_1 _<*a*_2 _< ... <*a*_ℓ _≤ *n*) is called a *subsequence structure *of **S**. **S***' *is also called a *k-reduced subsequence structure *of **S**, where *k *= |**S**| - |**S***'*|. For two structures **S **= () and **T **= (), the concatenated structure () is denoted by **S **∘ **T**. *R*·**S **denotes the structure **S **rotated by the rotation matrix *R*, *i.e.*, *R*·**S **= ().

 denotes the transpose of the vector  and *A*^*T *^denotes the transpose of the matrix *A*. *trace*(*A*) denotes the trace of the matrix *A*. || denotes the norm of the vector .  denotes the zero vector. ⟨*x*⟩ denotes the expected value of *x*. *P rob*() denotes the probability of the event .

### RMSD: Root Mean Square Deviation

The RMSD (root mean square deviation) [18-23] is the most widely-used geometric similarity measure between two sequences of 3-D coordinates. The RMSD between two 3-D coordinates sequences **S **= () and **T **= () is defined as the minimum value of(1)

over all the possible rotation matrices *R *and translation vectors . Note that the RMSD can be defined in any other dimensions by considering the above vectors and matrices in any *d *dimensions. Let *RMSD*(**S**, **T**) denote the minimum value, and let (**S**, **T**) and (**S**, **T**) denote the rotation matrix and the translation vector that minimizes (**S**, **T**).

Kabsch [20,21] proposed an efficient linear-time algorithm to compute *RMSD*(**S**, **T**), (**S**, **T**) and (**S**, **T**) (in 3-D space) as follows. If the rotation matrix *R *is fixed, (**S**, **T**) is known to be minimized when the centroid (center of mass) of *R*·**T **is translated to the centroid of **S **by the translation vector , regardless of what the rotation matrix *R *is. It means that (**S**, **T**) can be computed in linear time if we are given (**S**, **T**). Moreover, it also means that the problem of computing the RMSD can be reduced to a problem of finding *R *(*i.e.*, (**S**, **T**)) that minimizes (**S**, **T**) = , by translating both **S **and **T **so that both of their centroids are moved to the origin of the coordinates, which can be done in linear time. If both structures have been already translated so that both centroids are moved to the origin, we can compute (**S**, **T**) in linear time as follows [18,20,21]. Let *J *= . Clearly, *J *can be computed in *O*(*n*) time. Then (**S**, **T**) can be described as  - 2·*trace*(*R*·*J*), and *trace*(*R*·*J*) is maximized when *R *= *VU*^*T*^, where *U*Λ*V *is the singular value decomposition (SVD) of *J*. Thus (**S**, **T**) can be obtained from *J *in constant time, as *J *is a 3 × 3 matrix and the SVD can be computed in *O*(*d*^3^) time for a *d *× *d *matrix [33]. Note that there are degenerate cases where *det*(*V U *^*T*^) = -1, which means that *V U *^*T *^is a reflection matrix. See [18,19] for the details of the degenerate cases. Finally, we can compute the RMSD in linear time once we have obtained (**S**, **T**). In total, we can compute the RMSD in *O*(*n*) time.

### Random-Walk Model for Chain Molecules

The *random-walk model *(also called the *freely-jointed chain model*, or just the *ideal chain model*), is a very widely used simple model for analyzing behavior of chain molecules in molecular physics [29-32]. The model is also used for analyzing the computational time complexities of algorithms for protein structures [10]. In the model, we assume that the chain molecules can be considered as random walks. The model ignores many physical/chemical constraints, but it is known to reflect the behavior of real molecules very well. In fact, experiments in [10] showed high consistency between the experimental results obtained from the PDB database and the theoretical results deduced from the random-walk model.

Consider a chain molecule **S **= () of length *n *+ 1, in which the distance between any two adjacent atoms is fixed to some constant *r*. In the random-walk model, a bond between two adjacent atoms, *i.e.*, , is considered as a random vector that satisfies || = *r*, and  is considered to be independent from any other bond  (*j *≠ *i*). In the case of proteins, the distance between two adjacent *C*_*α *_atoms is fixed to 3.8Å. Note that we can let *r *= 1 by considering the distance between two adjacent atoms as the unit of distance.

### Shibuya's Lower Bound of the RMSD [10]

Let **U**^*left *^denote () and **U**^*right *^denote () for a structure **U **= (). Let *G*(**U**) denote the centroid of the structure **U**, *i.e.*, *G*(**U**) = . Let *F *(**U**) denote |*G*(**U**^*left*^) - *G*(**U**^*right*^)|/2, and let *D*(**S**, **T**) denote  for two structures such that |**S**| = |**T**|. Shibuya proved the following two lemmas in [10]:

**Lemma 1 **(**Shibuya **[10]) *D*(**S**, **T**) *is always smaller than or equal to RMSD*(**S**, **T**).

**Lemma 2 **(**Shibuya **[10]) *The probability Prob*(*D*(**S**, **T**) <*c*) *is in O*(*c*/), *where n *= |**S**| = |**T**|, *under the assumption that either ***S **or **T ***follows the random-walk model*.

Shibuya utilized the above lower bound *D*(**S**, **T**) for developing his breakthrough average-case linear time algorithm for searching substructures from 3-D databases without indels. Moreover, he showed that experimental results on the whole PDB database had very high consistency with Lemma 2. We will also utilize the above two lemmas for developing our average-case linear algorithm for a problem with indels, but our algorithm is different from the algorithms in [10].

## The *k*-Indel 3-D Substructure Search Problem

We focus on the following problem.

***k*-Indel 3-D Substructure Search Problem**: We are given a text structure **P **of size *N *and a query structure **Q **of size *m *(1 <*m *≤ *N*), both of which are represented by 3-D coordinates sequences of the residues. We are also given a constant positive real *c *and a positive integer *k *(*k *<*m*). The problem is to find all the positions *i *(1 ≤ *i *≤ *N *- *m *+ *k *+ 1) such that the RMSD between some *k'*-reduced subsequence structure of **Q **and some *k"*-reduced subsequence structure of **P **[*i*..*i *- *k' *+ *k" *+ *m *- 1] is at most *c*, for some non-negative integers *k' *and *k" *(*k' *+ *k" *≤ *k*, *k" *- *k' *≤ *N *- *m *- *i *+ 1).

If there exists some triple set {*i*, *k'*, *k"*} that satisfies the above condition, we say that **Q **matches with **P **[*i*..*i *- *k' *+ *k" *+ *m *- 1] with threshold *c *and (at most) *k' *+ *k" *indels. Usually, *c *is set to a constant proportional to the distance between two adjacent residue coordinates in the molecular structures. In the case of protein structures, *c *is often set to 1-2Å, while the distance between two adjacent *C*_*α *_atoms is 3.8Å. Structure databases usually contain more than one structure, but problems against the databases with multiple structures can be reduced to the above single-text problem by just concatenating all the structures into a single long text structure and ignoring matches that cross over the boundaries of two concatenated structures.

The special case of the problem where *k *= 0 has been well studied. If we directly apply the Kabsch's algorithm [20,21], the problem without indels can be solved in *O*(*Nm*) time. For the problem, Schwartz and Sharir [22] proposed an algorithm based on the fast Fourier transform technique that runs in *O*(*N *log *N*) time, which can be easily improved into an algorithm that runs in *O*(*N *log *m*) time [10]. Recently, Shibuya [10] proposed an average-case linear time algorithm, assuming that the text structures follow the random-walk model. He showed that the experimental results on the whole PDB database agrees with the theoretical analysis based on the random-walk model. But none of these algorithms considers any indels.

On the other hand, there have been almost no algorithmic study for cases *k *> 0, due to the difficulty of the problem, though the problem is very important. Any of the above algorithms for the case *k *= 0 cannot be applied to the *k *> 0 cases. (Note that our algorithm in this paper can be applied to the *k *= 0, but it could be less efficient than the algorithm in [10].) Moreover, the difficulty of the problem is not well known. In a later section, we will show that the problem is NP-hard, in case the dimension of the problem is arbitrary. According to the preliminaries section, the RMSD between two structures of size *m *can be computed in *O*(*m*) time. The possible number of subsequence structures to be compared in the *k*-indel 3-D substructure search problem is less than _2*m*+*k*_*C*_*k*_·*N*, which is in *O*(*Nm*^*k*^). Thus, our problem can be computed in *O*(*Nm*^*k*+1^) time, either in the worst-case analysis or in the average-case analysis. As far as we know, it is the best-known time complexity, and there have been known no algorithms other than the above straightforward algorithm. But it also means that the problem can be computed in polynomial time, in case the number of indels is bounded by some constant. In a later section, we will propose the first algorithm with better average-case time complexity, *i.e.*, *O*(*N*), for the above problem in case the number of the indels is at most some constant, which is a substantial improvement for the problem. Note that the worst-case time complexity of our algorithm is still the same as the above straightforward algorithm. Note also that our analysis of the average-case time complexity is based on the assumption that the text structure follows the random-walk model, like the analysis in [10]. We give no assumption on the query structures, but the same can be said in case we give the random-walk assumption on the query structures instead of the text structures.

## An NP-Hardness Result

Consider the following variant of the *k*-indel 3-D substructure search problem.

***k*-Indel Structure Comparison Problem**: We are given two structures **P **and **Q**, both of whose lengths are *n*. Find a *k*-reduced subsequence structure **P***' *of **P **and a *k*-reduced subsequence structure **Q***' *of **Q**, such that the RMSD between **P***' *and **Q***' *is at most some given threshold *c*.

It is trivial that the *k*-indel structure comparison problem is in the class NP, as the correctness of any instance can be checked in linear time. Moreover, it is also trivial that the *k*-indel 3-D substructure search problem is at least as difficult as the *k*-indel comparison problem in 3-D, and the *k*-indel 3-D substructure search problem is NP-hard if the *k*-indel structure comparison problem in 3-D is NP-complete. The two problems can be extended to the problems in any dimensional space. From now on, we show the *k*-indel structure comparison problem in arbitrary dimension is NP-complete, by reduction from the following *k*-cluster problem (or the densest *k*-subgraph problem), whose decision problem is known to be NP-complete [34].

***k*-Cluster Problem (Densest k-Subgraph Problem)**: Given a graph *G *= (*V, E*) and a positive integer *k *(*k *< |*V*|), find a size *k *subset of *V *such that the number of edges induced by the subset is the largest.

Let *V *= {*v*_1_, *v*_2_, ..., *v*_*n*_}. Consider an arbitrary subset *V' *= {*v*_*g*1_, *v*_*g*2_, ..., *v*_*gk*_} of *V*, where *g*_1 _<*g*_2 _< ... <*g*_*k*_, and let *x *be the number of edges induced by *V'*.

There must exist a sequence of points **P **= () in *n *- 1 dimensional space, such that  = *α *if {*v*_*i*_, *v*_*j*_} ∈ *E *and  = *β *if {*v*_*i*_, *v*_*j*_} ∉ *E*, where *α *and *β *are any constants that satisfy 0 <*α *<*β *< 2*α*. Let **Q **be a sequence of *n *zero vectors (, ..., ) in the same *n *- 1 dimensional space. Let **P**_*V' *_= (), and **Q**_*V' *_be a sequence of *k *zero vectors (, ..., ) in the *n *- 1 dimensional space.

It is well known that the translation of the two structures in 3-D is optimized when the centroids of the two structures are placed at the same position (*e.g.*, at the origin of the coordinates) [18,20], in computing the RMSD. It is also true in any dimensions *d*, which can be easily proved as follows. Consider two arbitrary *d*-dimensional structures **S **= () and **T **= (), and an arbitrary *d*-dimensional translation vector . Then the following equation holds:(2)

Thus the translation is optimized when . It means that the translation is optimized when the two structures are moved so that the centroids of the two structures are at the same position. From now on, we consider computing the RMSD between **P**_*V' *_and **Q**_*V'*_. It is trivial that the centroid of **Q**_*V' *_is at the origin of the coordinates, and moreover **Q**_*V' *_does not change its shape by any rotation, as all the vectors in **Q***V' *are zero vectors. Hence, we do not have to consider the optimization of the rotation for computing the RMSD between the two structures. Therfore we obtain the following equation:(3)

It means that *RMSD*(**P**_*V'*_, **Q**_*V'*_) is smaller if *x *is larger, as 0 <*α *<*β*. Thus we can obtain the answer of the decision problem of the *k*-cluster problem by solving the (*n *- *k*)-indel *n *- 1 dimensional structure comparison problem on the two structures **P **and **Q**. Hence the *k*-indel structure comparison problem in arbitrary dimensional space is NP-complete, and consequently we conclude that the *k*-indel substructure search problem in arbitrary dimensional space is NP-hard:

**Theorem 1 ***The k-indel substructure search problem in arbitrary dimensional space is NP-hard*.

## The New Average-Case Linear Time Algorithm for 3-D

### The Algorithm

To improve the performance of the algorithms for approximate matching of ordinary textual strings, we often divide the query into several parts and use them to filter out hopelessly dissimilar parts in the text [35]. For example, in case we want to search for textual strings with *k *indels, we can efficiently enumerate candidates of the matches by dividing the query into *k *+ 1 substrings and finding the exact matches of these divided substrings, as at least one of the divided substrings must exactly match somewhere in the text. In a similar way, we also divide the query 3-D structure into several substructures and use them to improve the query performance in our algorithm for the *k*-indel 3-D substructure search problem. Our strategy is very simple and is as follows: Our algorithm first divides the query into 3*k *+ 2 parts, and then enumerates candidates of the matches by filtering out text substructures without enough substructures seeming to be similar to the divided query substructures. Finally, our algorithm naively computes the RMSDs against each of the remaining candidates to check whether they are actually matches or not.

Before describing our algorithms in detail, we introduce the following lemma, on which our algorithm is based.

**Lemma 3 ***Consider a pair of two structures ***S **= () *and ***T **= (), *both of whose length is n. Let ***S***' *= () *be some subsequence structure of ***S**, *and let ***T***' *= (). *Then, RMSD *(**S***'*, **T***'*), ≤ ·*RMSD*(**S**, **T**).

**Proof: **According to the definition of the RMSD, the following inequality holds:(4)

In our algorithm, we divide the query **Q **of size *m *into 3*k *+ 2 equal-length substructures of size *m' *= ⌊*m*/(3*k *+ 2)⌋. Note that *k *is the number of maximum indels, which is considered to be a small constant. We call each substructure a 'divided substructure'. Let **Q**_*j *_denote the *j*-th divided substructure, *i.e.*, **Q **[(*j *- 1)*m' *+ 1.. *j*·*m'*]. Let **M **denote the remaining part **Q **[(3*k *+ 2)*m' *+ 1.. *m*]. (If *m *= (3*k *+ 2)*m'*, **M **is a zero-length structure.) Note that **Q**_1 _∘ **Q**2 ∘ ⋯ ∘ **Q**_3*k*+2 _∘ **M **= **Q**. Then the following lemma holds:

**Lemma 4 ***If Q matches with P *[*i*..*i *- *k' *+ *k" *+ *m *- 1] *with threshold c and k *= *k' *+ *k" indels, then at least *2*k *+ 2 *divided substructures ***Q**_*j *_= **Q**[(*j *- 1)*m' *+ 1.. *j*·*m'*] *of ***Q ***(among the *3*k *+ 2 *divided substructures) satisfy the following constraint (Constraint 1).*

**Constraint 1 ***There exists a substructure P *[ℓ..ℓ + *m' *- 1] *of P such that RMSD*(, *P *[ℓ..ℓ + *m' *- 1]) ≤ *c **and i *+ (*j *- 1)*m' *- *k *≤ ℓ ≤ *i *+ (*j *- 1) *m' *+ *k*.

**Figure 1:**
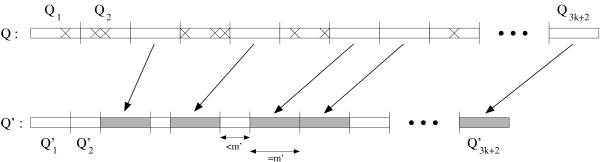
**Ungapped substructures**. There are at least 2*k *+ 2 pairs of subsequence structures  and  such that such that  = **Q**_*j *_and  is a substructure of **P**.

**Proof: **Suppose that **Q **matches with **P **[*i*..*i *- *k' *+ *k'' *+ *m *- 1] with threshold *c *and *k *= *k' *+ *k" *indels. Let **Q***' *and **P***' *denote the *k"*-reduced subsequence structure of **Q **and the *k"*-reduced subsequence structure of **P **[*i*..*i *- *k' *+ *k" *+ *m *- 1] respectively, such that *RMSD*(**Q***'*, **P***'*) ≤ *c*. Let  and **M***' *be 3*k *+ 3 substructures of **Q***' *such that  is a subsequence structure of **Q**_*i*_, **M***' *is a subsequence structure of **M**, and . Let *h*_*j *_(1 ≤ *j *≤ 3*k *+ 2) denote the first index of  in **Q***'*, and let *h*_3*k*+3 _denote the first index of **M***' *in **Q***' *(*i.e.*,  = **Q***' *[*h*_*j*_..*h*_*j*+1 _- 1]). Let **P***' *[*h*_*j*_..*h*_*j+1 *_- 1] (1 ≤ *j *≤ 3*k *+ 2). It is easy to see that there are at least 2*k *+ 2 pairs of 3*k *+ 2 subsequence structures  and  such that  = **Q**_*j *_and  is a substructure of **P **[*i*..*i *- *k' *+ *k" *+ *m *- 1] (1 ≤ *j *≤ 3*k *+ 2). We call these (at least 2*k *+ 2 pairs of) substructures 'ungapped substructures' (See Figure 1).

According to lemma 3, an inequality *RMSD*(, ) ≤ *c*· holds for ungapped substructures  and , as || = || = |**Q**_*j*_| = *m'*. If an ungapped structure  is the equivalent of **P **[ℓ..ℓ + *m' *- 1], it is easy to see that *i*+(*j *- 1)*m*' - *k *≤ ℓ ≤ *i*+(*j *- 1)*m' *+ *k*, as we allow only at most *k *indels. Hence, at least 2*k *+ 2 divided substructures **Q**_*j *_= **Q **[(*j *- 1)*m' *+ 1.. *j*·*m'*] (among the 3*k *+ 2 divided substructures) must satisfy Constraint 1.

Recall from Lemma 1 in the preliminaries section that *D*(**S**, **T**) provides a lower bound on the value of *RMSD*(**S**, **T**). This immediately yields the following lemma analogous to Lemma 4 for a somewhat weaker constraint (Constraint 2) which can be checked more efficiently than Constraint 1.

**Lemma 5 ***If some ***Q ***matches with ***P **[*i*..*i *- *k' *+ *k" *+ *m *- 1] *with threshold c and k *= *k' *+ *k" indels, then at least *2*k *+ 2 *divided substructures ***Q**_*j *_= **Q **[(*j *- 1)*m' *+ 1.. *j*·*m'*] *of ***Q ***(among the *3*k *+ 2 *divided substructures) satisfy the following constraint (Constraint 2)*.

**Constraint 2 ***There exists a substructure ***P **[ℓ..ℓ + *m' *- 1] *of ***P ***such that D *(, **P **[ℓ..ℓ + *m' *- 1]) ≤ *c*·*and i *+ (*j *- 1)*m' *- *k *≤ ℓ ≤ *i *+ (*j *- 1)*m' *+ *k*.

**Proof: **According to Lemma 4, at least 2*k *+ 2 divided substructures satisfy Constraint 1. Moreover, it is trivial that a divided substructure that satisfies Constraint 1 also satisfies Constraint 2, as an inequality *D*(**S**, **T**) ≤ *RMSD*(**S**, **T**) holds for any pair of same-length structures **S **and **T **by Lemma 1. Hence, at least 2*k *+ 2 divided substructure satisfy Constraint 2.

We call a divided substructure a 'hit substructure' for the position *i iff *it satisfies Constraint 2. Based on the above discussions, we propose the following simple algorithm for the *k*-indel 3-D substructure problem.

#### Algorithm

1. Enumerate all the positions *i *in **P **such that there are at least 2*k *+ 2 hit substructures for the position *i*, by computing all the *D*(**Q**_*j*_, **P **[*i*..*i *+ *m' *- 1]) values for all the pairs of *i *(1 ≤ *i *≤ *N *- *m' *+ 1) and *j *(1 ≤ *j *≤ 3*k *+ 2).

2. For each position *i *found in step 1, check the RMSDs between all the pairs of *k'*-reduced subsequence structure of **Q **and *k"*-reduced subsequence substructure of **P **[*i*..*i *+ *m *- *k' *+ *k" *+ *m *- 1] such that *k' *+ *k" = k *and *k" *- *k' *≤ *N *- *m *- *i *+ 1. If any one of the checked RMSDs is smaller or equal to *c*, output *i *as the position of a substructure similar to the query **Q**.

In the next section, we analyze the average-case time complexity of the algorithm.

### The Average-Case Time Complexity of the Algorithm

For each **Q**_*j *_(whether it is a hit substructure or not), we can compute *D*(**Q**_*j*_, **P **[*i*..*i *+ *m' *- 1]) for all *i *(1 ≤ *i *≤ *N *- *m' *+ 1) in total *O*(*N*) time, as *G*(**P **[*i*..*i *+ *m' *- 1]) (*i.e.*, the centroid of **P **[*i*..*i *+ *m' *- 1]) can be computed in *O*(*N*) time for all *i*. Thus, we can execute the step 1 of our algorithm in *O*(*k*^2^·*N*) time. Let *N' *denote the number of candidates enumerated in step 1 of our algorithm. As the number of pairs to check in step 2 for each position is less than _2*m*+*k*_*C*_*k *_(which is in *O*(*m*^*k*^)), and each RMSD can be computed in *O*(*m*) time, the computational complexity of step 2 is *O*(*N *'*m*^*k*+1^). In total, the computational complexity of the algorithm is *O*(*k*^2^·*N *+ *N 'm*^*k*+1^). In the worst case, the algorithm could be as bad as the naive *O*(*Nm*^*k*+1^)-time algorithm, as *N' *could be *N *at worst.

But, in the following, we show that ⟨*N'*⟩ is only in *O*(*N/m*^*k*+1^) and consequently the average-case time complexity of the algorithm is astonishingly *O*(*N*), under the assumption that **P **follows the random-walk model and *k *= *O*(1). According to Lemma 2 in the preliminaries section, the probability that a divided substructure **Q**_*i *_is a hit substructure for the position *i *is in *O*(*k*·*c*·) = *O*(*c*·*k*^2^/), under the random-walk assumption. Consider that the above probability can be bounded by *a*·*c*·*k*^2^/, where *a *is some appropriate constant. Then, the probability that at least 2*k *+ 2 of the 3*k *+ 2 divided substructures are hit substructures is *O*((*a*·*c*·*k*^2^/)^2*k*+2^·_3*k*+2_*C*_2*k*+2_), which is in *O*(*c*^2*k*+2^·*k*^5*k*+4^/*m*^*k*+1^). Thus ⟨*N'*⟩ is in *O*(*N*·*c*^2*k*+2^·*k*^5*k*+4^/*m*^*k*+1^), and the following lemma holds, considering that both *c *and *k *are small fixed constants.

**Lemma 6 **⟨*N'*⟩ *is in O*(*N/m*^*k*+1^), *under the assumption that k is a constant*.

Consequently the average-case time complexity of the step 2 of the above algorithm is only in *O*(*N*). More precisely, it is *O*(*c*^2*k*+2^·*k*^5*k*+4^·*N*), which means our algorithm is not so efficient for large *k*, but the time complexity is still linear if *k *is a constant. In conclusion, the total average-case time complexity of our algorithm is only *O*(*N*), under the assumption that **P **follows the random walk model. Note that the same discussion can be done if the query **Q**, instead of **P**, follows the random walk model. Thus we obtain the following theorem.

**Theorem 2 ***The total average-case time complexity of our algorithm is O*(*N*), *under the assumption that k is a constant and ***P ***follows the random walk model*.

## Conclusions

We considered the *k*-indel 3-D substructure search problem, in which we search for similar 3-D substructures from molecular 3-D structure databases, with consideration of indels. We showed that the same problem in arbitrary dimensional space is NP-hard. Moreover, we proposed an average-case linear time algorithm, under the assumption that the number of indels is bounded by a constant and the database structures follow the random-walk model. There are several open problems. First of all, the computational complexity of our problem restricted to 3-D space is still unknown. As for our algorithm, it would be very interesting to examine the efficiency of our algorithm against actual existing databases such as the PDB database. The average-case time complexity of our algorithm is *O*(*N*) for a database of size *N*, but its coefficient, *i.e.*, *c*^2*k*+2^·*k*^5*k*+4^, is very large (*c *is the threshold of the RMSD and *k *is the maximum number of indels, both of which we consider as constant numbers). It would be more practical if we could design algorithms with better coefficients. Another open problem is whether we can design a worst-case (deterministically) linear-time, or near linear-time algorithm for our problem, though no worst-case linear-time algorithm is known even for the no-indel case.

## Competing interests

The authors declare that they have no competing interests.

## Authors' contributions

TS designed and analyzed the average-case linear time algorithm, and mainly wrote this paper. TS, JJ and KS proved the NP-hardness of the problem in arbitrary dimensions. All the authors read and approved the final manuscript.
